# Mutations in *ATP13A2* (PARK9) are associated with an amyotrophic lateral sclerosis-like phenotype, implicating this locus in further phenotypic expansion

**DOI:** 10.1186/s40246-019-0203-9

**Published:** 2019-04-16

**Authors:** Rossella Spataro, Maria Kousi, Sali M. K. Farhan, Jason R. Willer, Jay P. Ross, Patrick A. Dion, Guy A. Rouleau, Mark J. Daly, Benjamin M. Neale, Vincenzo La Bella, Nicholas Katsanis

**Affiliations:** 1 ALS Clinical Research Center, Department of Biomedicine, Neuroscience and Advanced Diagnostics, University of Palermo, via G La Loggia 1, 90129 Palermo, Italy; 2 IRCCS Centro Neurolesi Bonino Pulejo, Palermo, Italy; 3 Center for Human Disease Modeling, Duke University Medical Center, Carmichael Building, 300 North Duke Street, Suite 48-118, Durham, NC 27701 USA; 4 MIT Computer Science and Artificial Intelligence Laboratory (CSAIL), Cambridge, MA USA; 5 The Broad Institute of MIT and Harvard, Cambridge, MA USA; 6 Analytic and Translational Genetics Unit, Center for Genomic Medicine, Massachusetts General Hospital, Boston, MA USA; 7 Stanley Center for Psychiatric Research, Broad Institute of MIT and Harvard, Cambridge, MA USA; 8 Program in Medical and Population Genetics, Broad Institute of MIT and Harvard, Cambridge, MA USA; 9 Montreal Neurological Institute, and Hospital, McGill University, Montréal, QC, Canada; 10 Department of Human Genetics, McGill University, Montreal, QC, Canada; 11 Institute for Molecular Medicine Finland, Helsinki, Finland

## Abstract

**Background:**

Amyotrophic lateral sclerosis [1] is a genetically heterogeneous neurodegenerative disorder, characterized by late-onset degeneration of motor neurons leading to progressive limb and bulbar weakness, as well as of the respiratory muscles, which is the primary cause of disease fatality. To date, over 25 genes have been implicated as causative in ALS with *C9orf72*, *SOD1*, *FUS*, and *TARDBP* accounting for the majority of genetically positive cases.

**Results:**

We identified two patients of Italian and French ancestry with a clinical diagnosis of juvenile-onset ALS who were mutation-negative in any of the known ALS causative genes. Starting with the index case, a consanguineous family of Italian origin, we performed whole-exome sequencing and identified candidate pathogenic mutations in 35 genes, 27 of which were homozygous. We next parsed all candidates against a cohort of 3641 ALS cases; only *ATP13A2* was found to harbor recessive changes, in a patient with juvenile-onset ALS, similar to the index case. In vivo complementation of *ATP13A2* using a zebrafish surrogate model that focused on the assessment of motor neuron morphology and cerebellar integrity confirmed the role of this gene in central and peripheral nervous system maintenance and corroborated the damaging direction of effect of the change detected in the index case of this study.

**Conclusions:**

We here expand the phenotypic spectrum associated with genetic variants in *ATP13A2* that previously comprised Kufor-Rakeb syndrome, spastic paraplegia 78, and neuronal ceroid lipofuscinosis type 12 (CLN12), to also include juvenile-onset ALS, as supported by both genetic and functional data. Our findings highlight the importance of establishing a complete genetic profile towards obtaining an accurate clinical diagnosis.

**Electronic supplementary material:**

The online version of this article (10.1186/s40246-019-0203-9) contains supplementary material, which is available to authorized users.

## Introduction

ATP13A2 is a lysosomal protein and a member of the P5-type subfamily of the P-type transport ATPases, implicated in the transport of cations and other substrates across membranes [2–5]. To date, mutations in *ATP13A2* have been associated with three distinct neurodegenerative conditions. Specifically, loss-of-function mutations were reported to underlie Kufor-Rakeb syndrome, a rare autosomal recessive disorder recognized as a juvenile-onset form of Parkinson disease [6, 7]. Kufor-Rakeb syndrome is characterized by levodopa-responsive parkinsonism with dementia and pyramidal tract degeneration. It was first described in a family originating from a small community living in Jordan [6]; it has since been reported in other families from different ethnicities [8–10] and can show phenotypic variability, even within the same family [8, 10]. Subsequent to the identification of *ATP13A2* as a causative gene for Kufor-Rakeb syndrome, a homozygous p.Met810Arg allele was reported in a Belgian family with a juvenile-onset neuronal ceroid lipofuscinosis [11, 12]. Affected individuals showed a complex phenotype, which included parkinsonism, a bulbar syndrome with dysphagia/dysarthria, spinocerebellar ataxia, intellectual deterioration, peripheral neuropathy, and pyramidal signs.

In addition to these pathologies, mutations in *ATP13A2* have also been reported in a Bulgarian family with juvenile-onset hereditary spastic paraplegia (SPG78), characterized by a progressive pyramidal and cerebellar degeneration and cognitive deficits [13]. Supranuclear gaze palsy and peripheral axonal motor sensory neuropathy were found in several affected members. Whole-exome sequencing and homozygosity mapping led to the identification of a p.Thr512Ile homozygous variant. These findings were supported by the discovery of homozygous (p.Gln122Ter) and compound heterozygous nonsense variants (p.Arg444Ter/p.Gln1135Ter) in SPG78 patients from a Serbian and a Bosnian family, respectively [13].

ATP13A2 belongs to the family of P-type ATPases that involves enzymes mediating the coupling of active substrate transport with the hydrolysis of ATP. P-type ATPases have been further subclustered in five classes (P1–P5) [14]. Of these, the class of P5-ATPases, in which ATP13A2 belongs, remains the most poorly characterized with no putative transported substrate identified to date. Overall, P5-ATPases have been reported to localize in the endoplasmic reticulum (ER) where they are involved in protein maturation and secretion, or they have been reported to be integral members of the endosomal/lysosomal membranes [15]. ATP13A2, in particular, has been shown to span the lysosomal and late endosomal membranes under physiological conditions, but to be retained in the ER when affected by damaging mutations [7]. From a biochemical standpoint, ATP13A2 shows a complex structure with ten transmembrane segments and four functional domains, including an actuator domain (A) and a phosphorylation domain (P1), the latter containing the P-type DKTGTLT motif [2, 4, 7]. In physiological conditions, ATP13A2 undergoes catalytic autophosphorylation at the D508 site on the conserved P-type motif [5, 16], leading to protection from heavy metal ions and α-synuclein toxicity [3, 16–18]. The actuator domain contains a phosphatase responsible for the dephosphorylation of the P1 domain.

ATP13A2 is highly expressed in the brain of both rodents and humans [4, 7, 18]. In the latter, high expression levels were found in the pyramidal neurons of the cerebral cortex and the neuromelanin-containing dopaminergic neurons of the substantia nigra [2, 18]. Furthermore, increased levels of ATP13A2 have been reported in pathological conditions involving Parkinson disease and Lewy Body disease [18]. Interestingly, a protective role of ATP13A2 on mitochondria dysfunction has also been demonstrated [2, 16, 19].

Together, the genetic findings in *ATP13A2* intimate a model where recessive, likely loss-of-function mutations drive a diverse spectrum of neurodevelopmental and neurodegenerative phenotypes. Here, we expand the pathology of ATP13A2 through the identification and functional dissection of recessive mutations in two patients with a juvenile-onset amyotrophic lateral sclerosis [1] compatible phenotype. The phenotype of these individuals bears similarities to SPG78, in terms of early-onset progressive upper and lower motor neuron degeneration with ataxia, axonal sensory-motor neuropathy, and intellectual impairment. Exome sequencing analysis revealed a hitherto unreported homozygous nonsense change, p.Glu613Ter, and a homozygous missense variant, p.Ile411Met, in patients of Italian and French origins, respectively. Consistent with the host of reported neuronal pathologies, knockdown of the sole *atp13a2* zebrafish ortholog showed the gene to be essential for the development and function of cerebellar and spinal motor neurons. Our data expand the genetic and clinical spectrum of the *ATP13A2*-associated diseases, and it reinforces the critical role of lysosomal and mitochondrial dysfunction in neurodegenerative processes.

## Materials and methods

### Patients

All subjects described in this study signed an informed consent. All patients and unaffected individuals from the four-generation pedigree reported in this study underwent a thorough psychological exam and genetic counseling. Whole blood was obtained from all affected individuals for the purpose of whole-exome sequencing analysis. All samples were collected after an informed consent was obtained and all experiments were performed in accordance with the World Medical Association Declaration of Helsinki. This study was approved by the Ethics Committees of the University of Palermo and the Montreal Neurological Institute and Hospital (IRB00010120). Additional ALS samples were used as described in Farhan et al., 2018, BioRxiv (https://www.biorxiv.org/content/10.1101/307835v2).

### Whole-exome sequencing analysis, variant detection, and variant validation

We extracted genomic DNA from whole blood from the affected proband (MA80) and his parents (MS54 and GL59) from family N01 and from the sporadic individual N0203, following standard procedures. Genomic samples were used to perform whole-exome sequencing using an Illumina Hiseq 2000 platform for 100 base pair (bp) pair-end reads. To ensure data quality, the following quality control metrics were applied: > 70% of reads aligned to target, > 99% target base covered at >20×, > 85% target base covered at >40×, and mean coverage of target bases >100×. For data filtering to retain candidate functional variants, we only considered variants with minor allele frequency (MAF) < 1% in public databases including dbSNP; 1000 Genomes; the National Heart, Lung and Blood Institute (NHLBI) Exome Sequencing Project (*n* = 6500 exomes); the Exome Aggregation Consortium (ExAC with *n* = 60,706 exomes); the Genome Aggregation Database (gnomAD with *n* = 123,136 exomes and *n* = 15,496 genomes); and the Atherosclerosis Risk in Communities cohort (ARIC; *n* = 2300 exomes) sequenced internally in BCM-HGSC. All de novo*,* homozygous, or compound heterozygous variants were visually confirmed through Integrated Genomics Viewer (IGV) analysis. Candidate de novo changes or variants segregating in an autosomal recessive manner in MA80 that passed visual confirmation through IGV and had an allelic balance of > 30% for the minor allele in each position evaluated were confirmed by Sanger sequencing on an ABI3730 sequencer.

### Surveying additional ALS datasets

To search for additional *ATP13A2* variants in ALS patients, we used data from Farhan et al., BioRxiv, as well as from the ALS Variant Server (AVS, http://als.umassmed.edu/) hosted by the University of Massachusetts Medical School (Worcester, MA) and the IRCCS Istituto Auxologico Italiano - Università degli Studi di Milano (Milan, Italy). We scanned these resources for homozygous missense, nonsynonymous and splice altering variants within 277 sporadic ALS cases, and 500 familial ALS cases available from AVS, as well as 3864 from the ALS Knowledge Portal (ALSKP, http://alskp.org/ 2019-02-23). To evaluate the frequency of these variants in additional controls, we used gnomAD, which contains genetic information from > 130,000 individuals; however, we focused on gnomAD (non-neuro *n* = 114,704 samples) as a subset of the ALS data from Farhan et al. have been deposited into gnomAD. We also used data from the ALZFORUM Healthy Exomes (HEX) dataset (468 individuals over the age of 60; www.alzforum.org/exomes/hex). All individuals within HEX are deceased; however, they were cognitively intact with no clinical diagnosis of neurodegenerative disease and were neuropathologically healthy at autopsy. Importantly, we attempted to use additional ALS data from Project MinE; however, data for *ATP13A2* or its alias gene names were not available.

### Zebrafish assays

The in vivo experimental work was carried out under protocols approved by the Institutional Animal Care and Use Committee, Duke University, following standard laboratory procedures. Two splice-blocking morpholinos (MO) against *atp13a2* were designed and obtained from Gene Tools, LLC (*atp13a2*_ex4: 5′-GGCAGGTGAGACTCACAGTTCATTG-3′ and *atp13a2*_ex6: 5′-CACATTAGACAGTACCTTGCTTTGC-3′). We injected 1 nl (6 ng) of diluted *atp13a2*_ex4 + ex6 MOs (DMO) into wild-type zebrafish embryos at the one- to two-cell stage. To produce capped human mRNA for the rescue experiments (100 pg injected), we cloned both wild-type and p.Gln613Ter *ATP13A2* into the pCS2+ vector and in vitro transcribed the cDNAs using the SP6 Message Machine kit (Ambion). To assess the integrity of the peripheral nervous system (PNS), we evaluated the morphology and extension of motor neurons from the notochord to the ventral portion of two-day *post* fertilization (dpf) larvae. Secondarily, we also evaluated the impact of *atp13a2* perturbations on the formation of the cerebellum in 3 dpf larvae. For both analyses, larvae were whole-mount stained with an antibody against acetylated tubulin (T7451, Sigma-Aldrich). Embryos were fixed in Dent’s fixative (80% methanol, 20% DMSO) overnight at 4 °C. After rehydration with decreasing series of methanol in PBS, they were washed with 100% PBS and permeabilized with 10 μg/ml proteinase K followed by post-fixation with 4% paraformaldehyde (PFA). Embryos were subsequently washed in PBS, and then in immunofluorescence buffer (0.1% Tween-20, 1% BSA in 1× PBS) for 10 min at room temperature. The embryos were then incubated in blocking buffer (10% fetal bovine serum (FBS), 1% BSA in PBS) for 1 h at RT. After two 10-min washes in IF buffer, embryos were incubated in primary antibody (1:1000 mouse antibody against acetylated tubulin) in blocking solution, overnight at 4 °C. After two additional 10-min washes in IF buffer, embryos were incubated in the secondary antibody (Alexa Fluor goat antibody against mouse IgG [A21207, Invitrogen], 1:1000) in blocking solution, for 1 h at RT. For each test, 50–100 embryos were scored. All experiments were performed blind to injection cocktail in duplicate. To calculate significance, the Pearson *χ*^2^ test was used.

## Results

### Clinical characterization of the index patient MA80

**Fig. 1:**
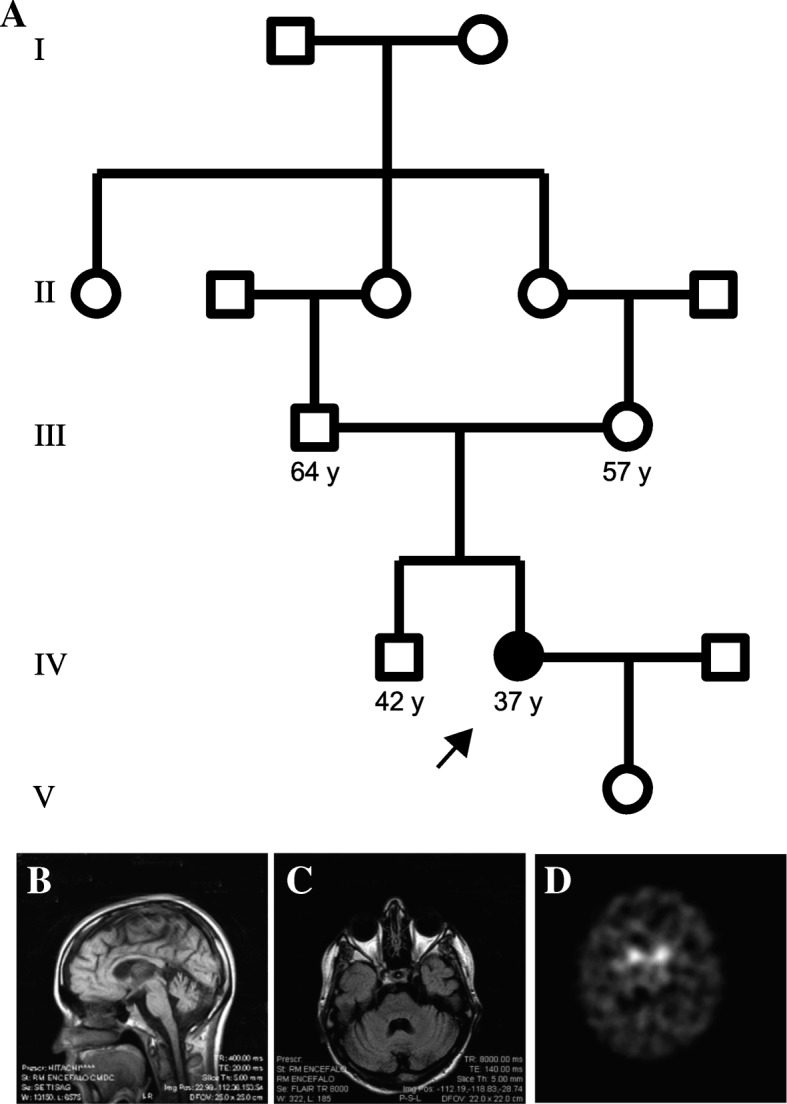
**a** Pedigree drawing of the five-generation family of Italian descent. Square symbols denote male and circles indicate female individuals. The filled circle in the fourth generation that is also highlighted with an arrow indicates the index case (MA80) that was exome sequenced. **b** Brain magnetic resonance imaging (MRI) analysis showing T1-weighted images of the sagittal and **c** axial representations of patient MA80, showing mild cortical, cerebellar, and vermian atrophy. **d** ^123^I-Ioflupane single-photon emission computerized tomography (SPECT) scan image of the basal ganglia, showing bilateral reduction in putamen uptake, associated with widespread non-specific cortical uptake

Proband MA80 (a 32-year-old female) was born to a consanguineous marriage with no prior family history of neurological conditions (Fig. 1a). She was referred to the Neurology Ward in Palermo, Italy, in March 2011, following the report of several months of weakness and rigidity in the right leg. The onset of the lower limb affectedness was initially subtle, with symptom worsening and later involvement of the contralateral leg as the disease progressed.

Upon neurological examination, mild intellectual disability (Wechler Adult Intelligence Score (WAIS) ≤ 45), dysphonia and pyramidal syndrome, which was more pronounced in the lower limbs with brisk reflexes, and bilateral Hoffmann and Babinski’s signs were reported. Gait was spastic-ataxic with a predominant spasticity. Primitive reflexes such as palmomental and suck reflexes could be elicited. The patient was not significantly dysphagic.

Extensive biochemical examinations using onconeural and anti-ganglioside antibodies, immunological assessment, and blood cell count analyses were negative. Cerebrospinal fluid (CSF) analysis showed no abnormalities and was negative for oligoclonal bands. Post-contrast brain imaging showed mild atrophy of the cerebellar vermis and of the cortex (Fig. 1b, c). Post-contrast spine magnetic resonance imaging (MRI) was within the normal range. Visual and acoustic evoked potentials were likewise normal. Concentric needle electromyography (EMG) reported neurogenic polyphasic motor unit potentials (MUP) in several muscles of the lower limbs, with neurophysiological evidence of motor axonal neuropathy in the lower limbs. Somatosensory evoked potentials and motor evoked potentials showed altered cortical complexes. Based on these signs, a diagnosis of autosomal recessive complex hereditary spastic paraplegia was made.

A follow-up clinical assessment in 2013 documented severe worsening of the spastic-ataxic gait, with the patient MA80 only being able to walk with the support of a cane. Moderate dysphagia primarily for liquids, sialorrhea, tongue atrophy with fasciculations, and brisk jaw reflex ensued. Her speech progressed to dysarthric and dysphonic. Eye movements were normal. Upper limb muscular tone was normal. The lower limbs showed marked spasticity (Modified Ashworth scale = 3) [20] with no additional apparent extrapyramidal signs or symptoms. Neurophysiologic studies documented an extension of neurogenic polyphasic MUP to the upper lower limbs, which included the bulbar muscles (i.e., genioglossus and masseter muscles). Though MRI examination did not reveal additional changes with respect to the imaging data obtained upon diagnosis (data not shown), assessment with ^123^I-Ioflupane DaT Scan showed bilateral reduced (~ 45%) striatal uptake (Fig. 1d).

The presence of clinical symptoms and signs involving both upper and lower motor neurons, i.e., a deterioration of the bulbar functions with a significant progression of the pyramidal syndrome, the evidence of lower limb axonal sensory-motor polyneuropathy, and a WAIS score indicative of mild intellectual disability represent a symptomatic constellation of a complex phenotype with prominent ALS-like characteristics.

Based on the clinical observations, a genetic evaluation of known ALS- and HSP-related determinants was performed (*SOD1*, *FUS*, *TARDBP*, *ALS2*, *C9orf72*, *SPG11*, *SPG14*, *SCA1*, and *SCA2*). No disease-causative changes were detected. To identify the underlying genetic determinant for the ALS-like phenotype in patient MA80, whole-exome sequencing analysis was undertaken.

### Whole-exome sequencing (WES) analysis of patient MA80

Whole-exome sequencing (WES) analysis of the trio involving patient MA80 and her unaffected parents focused on the identification of de novo and recessive variants. The analysis identified one de novo missense variant in *EPS8* (g.12:15784472C > A, c.1948C > A, p.Val650Phe) that was validated by Sanger. However, this allele has been reported in heterozygosity, in an individual of South Asian descent (rs754925249). Variants in an additional 27 genes segregated with the clinical phenotype in a homozygous recessive manner and an additional seven genes harbored compound heterozygous changes. Among those, we observed a homozygous variant, g.1:17318989G > A, c.1837G > A, p.Glu613Ter in *ATP13A2*, that was also Sanger confirmed. Given the prior association of *ATP13A2* with other motor neuron-affecting phenotypes like Kufor-Rakeb syndrome (MIM #606693), autosomal recessive spastic paraplegia type 78 (MIM #617225), and neuronal ceroid lipofuscinosis type 12 (CLN12; MIM #610513), this variant emerged as a strong candidate. *ATP13A2* p.Glu613Ter is a stop-gain variant in exon 17 (of 29). Screening for p.Glu613Ter in publicly available databases such as ExAC (*n* = 60,706 exomes), gnomAD (*n* = 138,632; 123,136 exomes and 15,496 genomes), NHLBI Trans-Omics for Precision Medicine (TOPMed; *n* = 62,784 genomes), HEX (*n* = 498 exomes), and DiscovEHR (*n*= > 50,000 exomes) did not reveal any heterozygous or homozygous carriers. Moreover, no individuals carried any homozygous protein-truncating variants (PTV) within these databases. This observation further supports that *ATP13A2* homozygous PTVs are ultra-rare or absent in the general population. In silico analysis of p.Glu613Ter yielded a CADD score of 35. Consistent with the pathogenic potential of this variant, RT-PCR from patient fibroblasts detected *ATP13A2* mRNA; cloning and sequencing of RT-PCR products showed to be homozygous for the mutant allele (data not shown), suggesting that the mutation might lead to the truncation of the protein; the current absence of a reliable antibody precludes the testing of the protein size or levels to test this hypothesis directly.

### Identification of a second ALS patient carrying a homozygous recessive *ATP13A2* variant

**Fig. 2:**
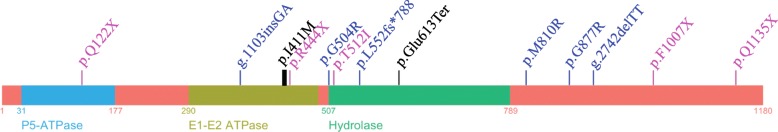
Schematic of ATP13A2 showing the hydrolase, as well as the P5- and E1-E2-ATPase protein domains. The mutations described in this study to be associated with an ALS-like phenotype are shown in black font. The variants in patients with Kufor-Rakeb syndrome are shown in blue, and the changes associated with hereditary spastic paraplegia are shown in magenta

To test the association of changes in *ATP13A2* with ALS-like phenotypes, we next mined data from the ALS Variant Server and from the ALSKP dataset (Fig. 2). We did not observe any homozygous variants within *ATP13A2* in the ALS Variant Server. However, we found an ALS-positive individual (patient 2) within the ALSKP dataset, who carries the missense variant g.1:17322954G > C, c.1233C > G, p.Ile411Met affecting the ATP13A2 E1-E2 ATPase domain, in homozygosity. Patient 2 is a male of French descent, who presented with spinal disease onset at 42 years of age, had no evidence of cognitive decline or frontotemporal dementia, and was negative for the *C9orf72* hexanucleotide (G4C2) expansion or for variants in any of the known ALS genes evaluated (*ALS2*, *ANG*, *ARHGEF28*, *ATXN2*, *C21orf2*, *CENPV*, *CHMP2B*, *DAO*, *DCTN1*, *FIG4*, *FUS*, *GRN*, *HNRNPA1*, *HNRNPA2B1*, *MAPT*, *MATR3*, *NEFH*, *NEK1*, *OPTN*, *PFN1*, *PNPLA6*, *PRPH*, *SETX*, *SIGMAR1*, *SOD1*, *SQSTM1*, *TAF15*, *TARDBP*, *TBK1*, *TUBA4A*, *UBQLN2*, *UNC13A*, *VAPB*, and *VCP*). ATP13A2 p.Ile411Met is absent from ExAC, NHLBI TopMed Bravo Browser, and DiscovEHR. When assessing the full gnomAD dataset (123,136 exomes and 15,496 genomes), we detected two individuals carrying ATP13A2 p.Ile411Met. The first was heterozygous for ATP13A2 p.Ile411Met and the second carried the variant in homozygosity. When focusing on the gnomAD (non-neuro) subset, we observe only one heterozygous individual (AF: 4.813e-6). We here report that patient 2, who is described in this study, is corresponding to the ATP13A2 p.Ile411Met homozygous individual in the gnomAD dataset, which harbors a large subset of ALS exomes (Farhan et al., BioRxiv, 2018, and personal communication). Finally, we integrated data from the Healthy Exomes (HEX) dataset comprising 468 individuals of > 60 years of age (Guerreiro et al., BioRxiv) but identified no heterozygous or homozygous individuals for ATP13A2 p.Ile411Met, p.Glu613Ter, or any other PTVs.

### In vivo complementation of ATP13A2 p.Glu613Ter in developing zebrafish embryos

**Fig. 3:**
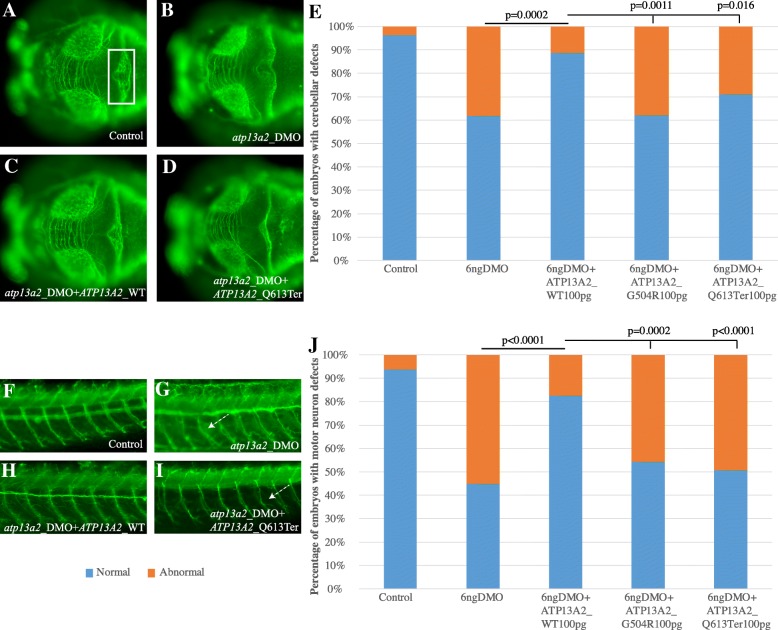
**a**–**d** Dorsal view of the brain of 3 days *post* fertilization (dpf) zebrafish larvae, visualized with an anti-acetylated tubulin antibody. The photographs of a control embryo (**a**), an embryo injected with morpholino (MO) oligonucleotides against exons 4 and 6 of the endogenous *atp13a2* (DMO; **b**), an embryo co-injected for DMO and wild-type (WT) human *ATP13A2* (**c**), and an embryo co-injected for DMO *ATP13A2* encoding the p.Glu613Ter change (**d**). The area of the cerebellum that was evaluated is highlighted with a white box in the control embryo (**a**). In embryos injected with DMO (**b**), significant disorganization of the axons that branch in the midline of the cerebellum is noted. The cerebellar phenotype was rescued by co-injection of DMO with WT human *ATP13A2* (**c**), but not with DMO + *ATP13A2* p.Glu613Ter (**d**) or DMO + *ATP13A2* p.Gly504Arg (data not shown), suggesting that these represent loss-of-function alleles. **d** Quantification of the percentage of embryos with cerebellar defects. **f**–**i** Lateral view of the peripheral nervous system (PNS) of two dpf embryos, showing the motor neurons that extend from the notochord dorsally to innervate the myotomes ventrally. The photographs of a control embryo (**f**), an embryo injected with DMO (**g**), DMO + *ATP13A2* WT (**h**), and DMO + *ATP13A2* p.Glu613Ter (**i**). In embryos injected with DMO (**g**), motor neurons deviate from the normal dorso-ventral path (white arrows). The phenotype was rescued by co-injection of DMO with *ATP13A2* WT (**h**), but not with DMO + *ATP13A2* p.Glu613Ter (**i**). **j** Quantification of the percentage of embryos with cerebellar defects. For both in vivo complementation assays, statistical significance was determined using a *χ*^2^ test

To obtain biological evidence for the causality of the nonsense variant (ATP13A2 p.Glu613Ter) identified in patient MA80, we developed an in vivo surrogate zebrafish model. A reciprocal BLAST search identified *atp13a2* as the sole zebrafish ortholog (67% similarity and 52% identity). We combinatorially used two morpholino antisense oligonucleotides (*atp13a2*_ex4 and *atp13a2*_ex6) to knock down the endogenous zebrafish expression of *atp13a2* (Additional file 1: Figure S1). Given the established role of *atp13a2* in the development and function of both the central and peripheral nervous systems, we evaluated the impact of *atp13a2* suppression on cerebellar integrity and motor neuron morphology. Suppression of *atp13a2* induced dose-sensitive defects in both the formation of the cerebellum, as well as the extension of motor neurons from the notochord (Fig. 3). Both phenotypes were specific, as supported by the phenotypic rescue observed when co-injecting embryos with wild-type human *ATP13A2* (*p* = 0.0002 for cerebellar integrity and *p* < 0.0001 for the motor neuron assay; Fig. 3). Next, we asked whether the discovered p.Glu613Ter variant harbored residual functional activity that would be sufficient to rescue the morphant phenotypes, similar to the wild-type human *ATP13A2* mRNA. To gain confidence in the assessment of the functional direction of effect through our complementation assay, we used a construct containing the p.Gly504Arg substitution, a bona fide pathogenic allele that causes Kufor-Rakeb syndrome [21], as a positive control. Testing for rescue of either cerebellar integrity or motor neuron extension showed consistently that the embryos co-injected with mutant human mRNA (p.Glu613Ter or p.Gly504Arg) were indistinguishable from morphants, corroborating the pathogenicity of p.Gly504Arg and suggesting that p.Glu613Ter represents a loss-of-function allele (*p* = 0.37 from MO and *p* = 0.016 from MO + WT for the cerebellar integrity assay; *p* = 0.62 from MO and *p* < 0.0001 from MO + WT for the motor neuron assay; Fig. 3). Overexpression of either wild-type or mutant *ATP13A2* did not induce any identifiable pathology in injected embryos arguing against these mRNAs having toxic effects (Additional file 1: Figure S2).

## Discussion

To date, mutations in *ATP13A2* have been associated with three overlapping yet distinct autosomal recessive disorders: Kufor-Rakeb syndrome (OMIM 606693), spastic paraplegia 78 (SPG78, OMIM 617225), and juvenile-onset neuronal ceroid lipofuscinoses (CLN12; 22388936). Here, we expand the phenotypic spectrum associated with mutations in *ATP13A2*, by reporting two cases with a juvenile-onset recessive ALS-like phenotype. The phenotypic mimicry of *ATP13A2* predominantly focuses in the manifestation of motor neuropathy, a symptom that appears to be cardinal in the presence of loss-of-function *ATP13A2* recessive variants. Despite the convergence of the disorders associated with such defects on motor neuron dysfunction, additional central nervous system defects determine the distinction of discrete clinical entities. Namely, Kufor-Rakeb syndrome is predominantly characterized by dementia and extrapyramidal parkinsonian syndrome; SPG78 by cognitive, pyramidal and cerebellar defects; CLN12 by spinocerebellar ataxia and cognitive decline; and the ALS-phenotype described here by intellectual disability, prominent upper and lower motor neuron degeneration, and cerebellar and cerebral atrophy.

To corroborate that the juvenile-onset ALS phenotype in the index case was due to the truncating variant (p.Glu613Ter) identified in *ATP13A2*, we generated a surrogate model to establish the variant direction of effect. We prioritized the functional testing of the *ATP13A2* variant predominantly due to its known association with neurodegenerative disorders and neuropathy. Our functional data confirmed that p.Glu613Ter is a loss-of-function allele that leads to the abolishment of protein function (likely by generating a truncated protein product) and hence the manifestation of defects in the formation of the cerebellum and the motor neurons. In addition to the truncating variant in *ATP13A2*, patient MA80 also carries a de novo missense variant in *EPS8* (p.Val650Phe). *EPS8* is not known to be associated with any known disorders in humans or model organism systems. Though the exact role of this gene remains unclear and it has been reported that only de novo variants are more likely to be functionally detrimental to protein function when absent from population databases [22], we cannot exclude the possibility that this allele might also be relevant to the patient’s clinical phenotype. In addition to the recessive changes in *ATP13A2*, the proband harbored recessive alleles in an additional 32 genes (Table 1). We cannot discount the possibility that some of these candidates might contribute to disease penetrance and expressivity. As such, elucidation of the impact of each of these candidates necessitates thorough functional interrogation through relevant surrogate models.

**Table 1:** Variants segregating with the clinical phenotype in patient MA80

Gene	Chromosomal position	Reference Allele	Alternate allele	Amino acid change	rs ID	Minor allele frequency (MAF)	MA80 zygosity	Unaffected sibling zygosity	OMIM disease association
De novo									
*EPS8*	12:15784472	C	A	p.Val650Phe	n/a	0%	Het	Hom ref	n/a
Autosomal recessive: homozygous									
*ATP13A2*	1:17318989	G	A	p.Gln613Ter	n/a	0%	Hom	Het	#606693
*PAH*	12:103249093	C	A	p.Gly176Val	rs74486803	0.015%	Hom	Het	#261600
*PRDM2*	1:14106485	G	A	p.Arg732Lys	n/a	0%	Hom	Het	n/a
*LUZP1*	1:23420658	C	T	p.Ala33Thr	n/a	0%	Hom	Het	n/a
*TRIM63*	1:26385068	G	A	p.Thr215Met	n/a	0%	Hom	Het	n/a
*AHDC1*	1:27877284	A	G	p.Val448Ala	n/a	0%	Hom	Het	n/a
*ZC3H12D*	6:149772097	G	C	p.Pro436Ala	rs367621958	0.17%	Hom	Het	n/a
*ZC3H12D*	6:149772499	G	C	p.Gly302Ser	n/a	0%	Hom	Het	n/a
*GALNT4*	12:89919749	G	A	p.Pro62Ser	n/a	0%	Hom	Het	n/a
*HOMEZ*	14:23755161	T	G	p.Met1Leu	n/a	0%	Hom	Hom ref	n/a
*ATP10A*	10:25962005	C	T	p.Val550Met	n/a	0%	Hom	Het	n/a
*CC2D1B*	1:52821892	G	A	p.His680Tyr	rs141717834	0.41%	Hom	Hom ref	n/a
*APHA2*	1:16475050	C	T	p.Ala216Thr	rs143736427	0.02%	Hom	Het	n/a
*EIF4G3*	1:21268143	T	C	p.Ile446Val	rs141254078	0.1%	Hom	Het	n/a
*HTR1D*	1:23519593	G	A	p.Arg374Trp	rs147605856	0.06%	Hom	Het	n/a
*NUDC*	1:27267996	C	G	p.Arg70Gly	rs140994074	0.02%	Hom	Het	n/a
*PPIL4*	6:149862510	T	C	p.Ile66Val	rs115372734	0.38%	Hom	Het	n/a
*LRP11*	6:150147424	C	T	p.Gly442Arg	rs9478144	0.68%	Hom	Het	n/a
*UBN2*	7:138943330	G	C	p.Glu254Gln	Rs202078043	0.06%	Hom	Het	n/a
*LUC7L2*	7:139102335	A	C	p.Glu284Asp	Rs118183173	0.6%	Hom	Het	n/a
*EPHB6*	7:142562055	C	T	p.Ser166Phe	Rs192960264	0.14%	Hom	Het	n/a
*SSPO*	7:149514822	A	T	p.Asn3792Tyr	Rs141999995	0.7%	Hom	Het	n/a
*FAM189A1*	15:29421059	A	C	p.Ser312Arg	Rs200682124	0.18%	Hom	Het	n/a
*DUOX2*	15:45403699	C	T	p.Gly200Arg	Rs2467827	0%	Hom	Het	n/a
*VSIG10L*	19:51837487	C	T	p.Val793Ile	Rs147142812	0.18%	Hom	Het	n/a
*RDH13*	19:55556574	G	C	p.Phe288Leu	Rs56125820	0.7%	Hom	Het	n/a
*RALY*	20:32664865	–	CAG	p.Ala214insAlaSer	Rs10649600	0%	Hom	Het	n/a
Autosomal recessive: compound heterozygous									
*TTF2*	1:117635401	G	A	p.Glu952Lys	rs41306197	0.76%	Het	Hom ref	n/a
	1:117635504	G	A	p.Gly986Asp	rs143131893	0.05%	Het	Hom ref	n/a
*DDX59*	1:200619614	C	T	p.Arg418His	rs78882239	0.21%	Het	Hom ref	n/a
	1:200635618	C	G	p.Ser84Thr	rs146060105	0.34%	Het	Hom ref	n/a
	1:200635721	C	G	p.Ala50Pro	rs150913822	0.34%	Het	Hom ref	n/a
*BSN*	3:49699108	G	A	p.Gly3277Glu	n/a	0%	Het	Hom ref	n/a
	3:49700533	G	A	p.Gly3648Ser	n/a	0%	Het	Hom ref	n/a
*ADAMTS9*	3:64526863	C	T	p.Arg1810His	rs149060903	0.04%	Het	Hom ref	n/a
	3:64672566	G	C	p.Thr65Arg	rs192420947	0.01%	Het	Hom ref	n/a
*HECTD1*	14:31602546	T	C	p.Met1274Val	n/a	0%	Het	Hom ref	n/a
	14:31602838	G	C	p.Gln1208Glu	n/a	0%	Het	Hom ref	n/a
*PAPLN*	14:73717654	G	A	p.Glu169Lys	n/a	0%	Het	Het	n/a
	14:73729154	G	A	p.Arg754His	rs200118136	0.06%	Het	Hom ref	n/a
*LOC100653515*	17:76888077	T	C	p.His170Arg	n/a	0%	Het	Het	n/a
	17:76888155	C	T	p.Arg144Pro	n/a	0%	Het	Hom ref	n/a

From a population genetics standpoint, we were able to provide further credence for the causality of *ATP13A2* in juvenile-onset ALS, by identifying a second individual with recessive truncating alleles in this gene. As a caution, we would like to highlight that given the deposition of individuals that have received ALS, Alzheimer disease, psychosis, bipolar, or schizophrenia diagnoses into gnomAD, the enrichment of specific alleles might be skewed when comparing an individual with a neurological diagnosis against the whole dataset. To overcome this limitation, each neurological case should only be compared against individuals with no neurological symptoms (“non-neuro”).

The mechanism through which mutations in the same gene might lead to the four distinct clinical phenotypes remains unclear. No mutation hot spots have been recognized, nor do the mutations cluster in specific domains of the protein. As such, we cannot draw genotype-phenotype correlations. To explain the broad clinical spectrum caused by *ATP13A2* mutations, modifiers or differential genetic burden in each reported case present attractive hypotheses that remain to be further tested. Specifically, though recessive variants in *ATP13A2* are necessary to cause neuronal dysfunction, they might not be sufficient. Instead, changes in other genes encoding for proteins that are either directly functionally linked to ATP13A2 such as molecules transported by this ATPase, or indirectly through their participation in lysosomal function, might account for the differential phenotypic manifestation. To explore these hypotheses, it is important to establish a complete genetic profile of the affected individuals for all genes and irrespective of whether the variants identified fulfill the criteria for clinical manifestation (i.e., homozygosity in suspected clinical disorders). A similar paradigm was reported in a comprehensive exome study, reporting that 4.9% of screened patients had diagnostic alleles in more than one gene, with each gene contributing distinct clinical features [23].

## Conclusions

The need to obtain detailed genotype information on every screened patient, either through exome or genome studies, becomes increasingly more recognized to make informed genotype-phenotype correlations. Here, we provide a comprehensive list of all variants in addition to *ATP13A2* that could serve as either drivers or phenotypic modifiers, ultimately leading to the presentation of a clinical picture that resembles juvenile-onset ALS. These data expand the phenotypic spectrum of causality for this locus and suggest that intersection of the lists of such “secondary” candidates and evaluation of the genetic burden across relevant genes such as lysosomal or neuronal specific proteins will be a critical and informative next step.

## Additional file

Additional file 1: **Figure S1.** a Schematic of the endogenous zebrafish *atp13a2* ortholog. The exons are shown are rectangles and the introns as horizontal lines. The splice junctions targeted with antisense morpholino oligonucleotides (*atp13a2*_3x4_SB and *atp13a2*_3x6_SB) are shown with red asterisks. b Gel images showing the efficiency of the *atp13a2*_3x4_SB and *atp13a2*_3x6_SB morpholinos. The first lane in each gel photograph shows amplicons from the respective loci flanking the targeted sequences, with no aberrations observed. In the embryos injected with morpholino oligonucleotides, aberrant bands were evident, showing that the morpholinos were efficient in knocking down the expression of endogenous *atp13a2*. Figure S2 a Bar graph showing the percentage of embryos displaying motor neuron defects upon overexpression of either wild-type or mutant human *ATP13A2* mRNA. We observed no statistically significant difference in the number of control versus injected embryos for any of the mRNAs tested, suggesting that both the wild-type and mutant transcripts (*ATP13A2* WT, *ATP13A2* p.Gly504Arg, *ATP13A2* p.Glu613Ter) do not induce dominant negative effects. (PDF 13488 kb)
