# Supplementary figures and images for: Mutations in ATP13A2 (PARK9) are associated with an amyotrophic lateral sclerosis-like phenotype, implicating this locus in further phenotypic expansion
Source: Hum Genomics. 2019 Apr 16;13:19. doi: 10.1186/s40246-019-0203-9 (PMC6469102; doi:10.1186/s40246-019-0203-9)

**A**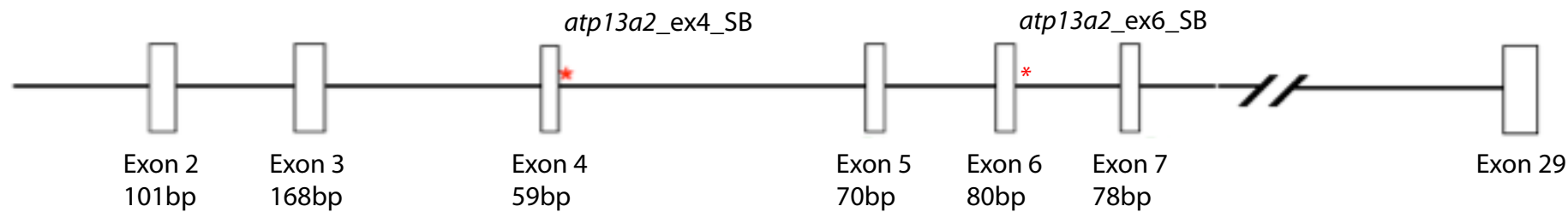**B**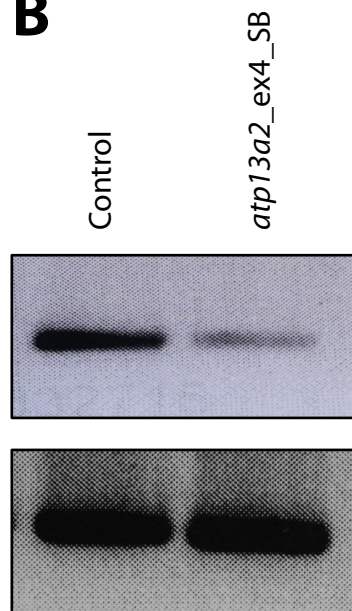**C**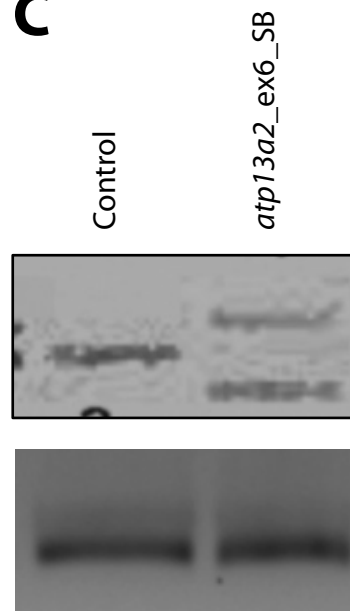

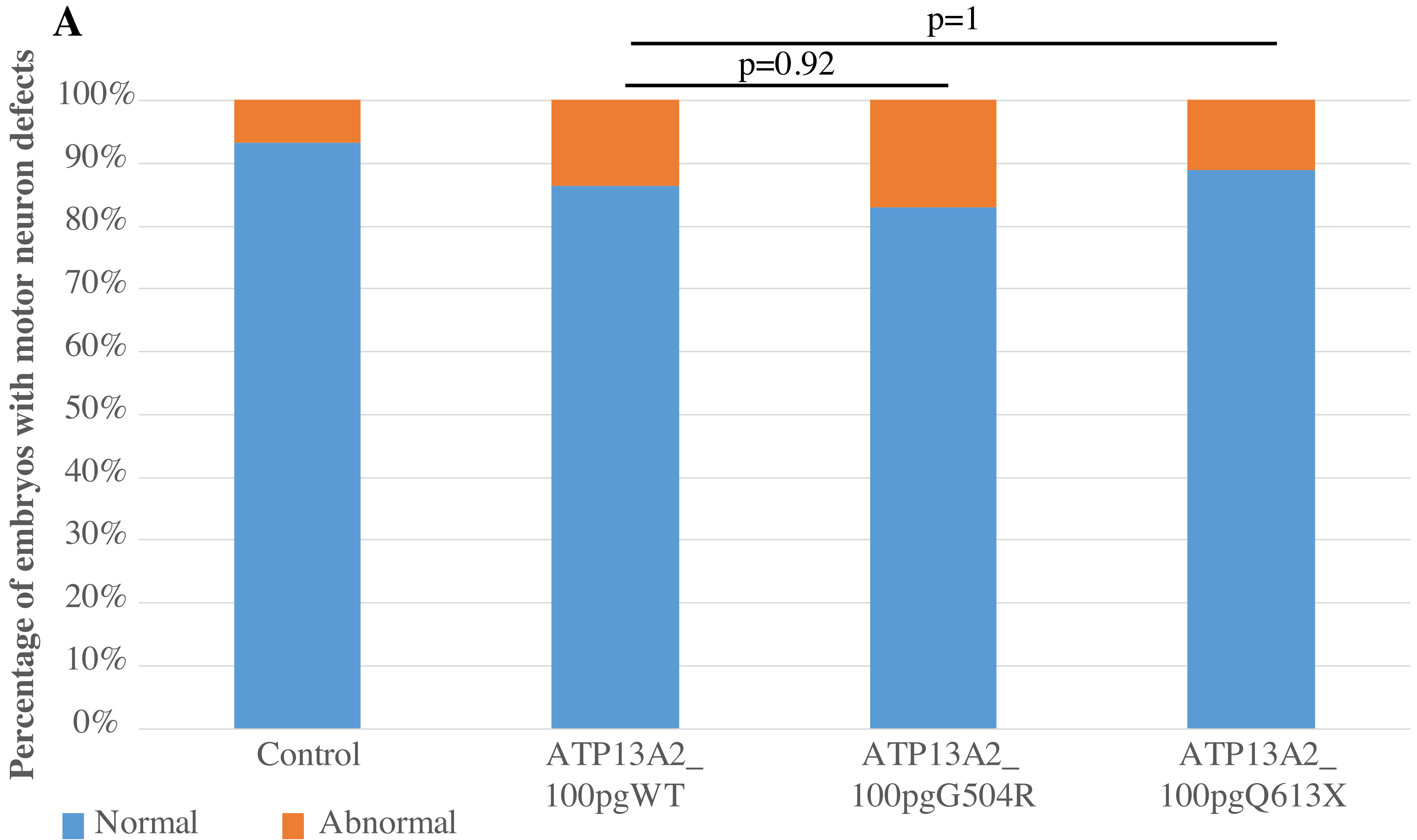

Supplement: Supplementary file 1 — Figure S1. a Schematic of the endogenous zebrafish atp13a2 ortholog. The exons are shown are rectangles and the introns as horizontal lines. The splice junctions targeted with antisense morpholino oligonucleotides (atp13a2_3x4_SB and atp13a2_3x6_SB) are shown with red asterisks. b Gel images showing the efficiency of the atp13a2_3x4_SB and atp13a2_3x6_SB morpholinos. The first lane in each gel photograph shows amplicons from the respective loci flanking the targeted sequences, with no aberrations observed. In the embryos injected with morpholino oligonucleotides, aberrant bands were evident, showing that the morpholinos were efficient in knocking down the expression of endogenous atp13a2. Figure S2 a Bar graph showing the percentage of embryos displaying motor neuron defects upon overexpression of either wild-type or mutant human ATP13A2 mRNA. We observed no statistically significant difference in the number of control versus injected embryos for any of the mRNAs tested, suggesting that both the wild-type and mutant transcripts (ATP13A2 WT, ATP13A2 p.Gly504Arg, ATP13A2 p.Glu613Ter) do not induce dominant negative effects. (PDF 13488 kb) [file 40246_2019_203_MOESM1_ESM.pdf]
